# Newcastle Disease Virus Inhibits the Proliferation of T Cells Induced by Dendritic Cells *In Vitro* and *In Vivo*

**DOI:** 10.3389/fimmu.2020.619829

**Published:** 2021-02-23

**Authors:** Fu Long Nan, Wei Zheng, Wen Long Nan, Tong Yu, Chang Zhan Xie, He Zhang, Xiao Hong Xu, Cheng Hui Li, Zhuo Ha, Jin Yong Zhang, Xin Yu Zhuang, Ji Cheng Han, Wei Wang, Jing Qian, Guan Yu Zhao, Zhuo Xin Li, Jin Ying Ge, Zhi Gao Bu, Ying Zhang, Hui Jun Lu, Ning Yi Jin

**Affiliations:** ^1^ College of Veterinary Medicine, College of Animal Science, Jilin University, Changchun, China; ^2^ Institute of Military Veterinary Medicine, Academy of Military Medical Sciences, Changchun, China; ^3^ The 964Hospital of the PLA Joint Logistics, Changchun, China; ^4^ China Animal Health and Epidemiology Center, Qingdao, China; ^5^ Agricultural College, Yanbian University, Yanji, China; ^6^ Academician Workstation, Changchun University of Chinese Medicine, Changchun, China; ^7^ Institute of Veterinary Medicine, Jiangsu Academy of Agricultural Sciences, Nanjing, China; ^8^ State Key Laboratory of Veterinary Biotechnology, Harbin Veterinary Research Institute, Chinese Academy of Agricultural Sciences, Harbin, China

**Keywords:** Newcastle disease virus, dendritic cells, antigen presentation, phenotypic maturation, immunosuppression, proliferation of T cells

## Abstract

Newcastle disease virus (NDV) infects poultry and antagonizes host immunity *via* several mechanisms. Dendritic cells (DCs) are characterized as specialized antigen presenting cells, bridging innate and adaptive immunity and regulating host resistance to viral invasion. However, there is little specific knowledge of the role of DCs in NDV infection. In this study, the representative NDV lentogenic strain LaSota was used to explore whether murine bone marrow derived DCs mature following infection. We examined surface molecule expression and cytokine release from DCs as well as proliferation and activation of T cells *in vivo* and *in vitro* in the context of NDV. The results demonstrated that infection with lentogenic strain LaSota induced a phenotypic maturation of immature DCs (imDCs), which actually led to curtailed T cell responses. Upon infection, the phenotypic maturation of DCs was reflected by markedly enhanced MHC and costimulatory molecule expression and secretion of proinflammatory cytokines. Nevertheless, NDV-infected DCs produced the anti-inflammatory cytokine IL-10 and attenuated T cell proliferation, inducing Th2-biased responses. Therefore, our study reveals a novel understanding that DCs are phenotypically mature but dysfunctional in priming T cell responses during NDV infection.

## Introduction

Newcastle disease (ND) is a highly contagious disease transmitted *via* the respiratory tract. The spread of ND results in great economic losses to the poultry industry worldwide. The causative agent of ND is Newcastle disease virus (NDV), a member of the family *Paramyxoviridae*, genus *Avulavirus*. The virus has a negative-sensed, single stranded and non-segmented RNA genome which encodes six structural proteins. NDV strains are classified as velogenic, mesogenic, or lentogenic strains. It has been reported that NDV suppresses the innate immune response through V protein-mediated MDA5, phospho-STAT1 and MAVS degradation (1, 2). NDV is reported to have distinct influences on dendritic cells (DCs) during the early stage of NDV infection, including activation of the DCs and cross-priming of naïve T cells into tumor-specific T cells (3, 4). However, another study indicated that infection with a velogenic NDV strain could stimulate an extrinsic apoptosis pathway in DCs, resulting in inhibition of CD4^+^ T cell proliferation (5). Hence, the full impact of viral infection, particularly on induction of adaptive immunity, has not been extensively evaluated.

DCs are professional antigen presenting cells (APCs) and function as messengers between innate and adaptive responses (APC to T cells) (6). Immature DCs capture antigens such as invading microbes or injured host cells from the peripheral tissue and activate other DCs to be matured, resulting in up-regulation of costimulatory molecules including CD80, CD86, CD40, and MHC, as well as several DC associated cytokines (7). Conversely, mature DCs (mDCs) display reduced antigen uptake ability, downregulated surface receptors such as DC-SIGN and CCR5, and present antigen efficiently to T cells (8, 9). Furthermore, mDCs upregulate the expression of CCR7, which mediates direct DC migration to draining lymph nodes through the CCR7-CCL19/CCL21 axis (10).

Paramyxoviruses suppress antigen presentation by DCs *via* multiple mechanisms to enhance viral proliferation. For example, measles virus (MV) and respiratory syncytial virus (RSV) alter the expression of costimulatory molecules, contributing to immune dysfunction (11–13). Furthermore, enhanced apoptosis triggered by MV and higher expression of IL-10 in DC/T-cell co-cultures also result in impeded T cell responses (14, 15). IL-10, induced by cytomegalovirus (MCMV), is an anti-inflammatory cytokine which exerts suppressive effects that regulate the virus-host balance to the benefit of the virus (16). Although infection with NDV triggers a strong innate immune response from DCs (17), the immunostimulatory properties of infected DCs which prime naïve T cells remain ill-defined. To clarify how NDV regulates antigen presentation of DCs, we investigated the maturation of DCs upon infection and its capacity to prime T cells both *in vitro* and *in vivo*. This provided a novel understanding of the role of NDV infected DCs in regulating the nature of innate-adaptive crosstalk.

## Materials and Methods

### Virus and Cells

The NDV lentogenic LaSota strain was rescued *via* reverse genetics (18) and propagated in 9-day-old specific pathogen free (SPF) embryonated chicken eggs. Virus was then titrated using BSR T7/5 cells and the concentration calculated using the method described by Reed and Muench (19).

Six-week-old female C57BL/6 mice were purchased from Beijing Vital River Lab Animal Technology Co., Ltd. Murine bone marrow derived DCs (BMDCs) were isolated from bone marrow and cultured in RPMI 1640 medium with 10% fetal bovine serum (Gibco, Carlsbad, CA, USA), 20 ng/ml rmGM-CSF, and 20 ng/ml rmIL-4 (R&D Systems, Minneapolis, MN, USA) at 37°C for 7 days. In subsequent experiments, 7-day cultured DCs were divided and sub-cultured at 10^6^ cells per well in 6-well plates. These imDCs were then treated with PBS (mock), NDV, or heat-inactivated NDV (HI) for 48 h.

### Flow Cytometry

To determine mature molecular marker expression on the surface after infection with NDV, cells were harvested at 48 hpi and stained with FITC or APC-labeled CD11c, PE-labeled CD80, MHC-I, MHC-II, CCR5, or CCR7, PE or PE-Cy5-labeled CD86 and PE or FITC-labeled CD40 antibodies (eBioscience, San Diego, CA, USA). After staining, maturity of infected DCs was determined by proportions of cells expressing markers (%) using flow cytometry.

Detection of apoptosis was performed using the Annexin V Assay Kit (Thermo Fisher Scientific, Waltham, MA, USA) according to the manufacturer’s protocol. Following infection with NDV, DCs or T cells were collected and incubated with CD11c-APC or CD3-PE-Cy5 antibodies, and then labeled with Alexa Fluor^®^ 488 annexin V and PI working solution. Thereafter, apoptosis of cells induced by NDV was analyzed by flow cytometry.

For intracellular cytokine staining, lymphocytes or DCs were pretreated with 2 mM Brefeldin A (to inhibit intracellular protein transport) for 4 h. After harvesting, cells were stained with CD11c-APC or CD3-PE-Cy5 and CD4-FITC/CD8-FITC antibodies. Next, cells underwent intracellular fixation and permeabilization and were subsequently stained with IL-10-PE, IFN-γ-PE, or IL-4-PE antibodies.

### Cytokine Production Measured by ELISA

Cell-free supernatants of infected DCs or co-cultures were collected at 48 h to measure secretion of IFN-α (Thermo Fisher Scientific, Waltham, MA, USA), IFN-β, IFN-γ, TNF-α, IL-10, and IL-12p70 (R&D Systems, Minneapolis, MN, USA) using ELISA kits according to the manufacturers’ instructions.

### Antigen Uptake Assays

Detection of antigen uptake by DCs was performed as described (20). 10^6^ DCs were treated with 1 mg/ml FITC-dextran (MW 40,000, Sigma-Aldrich, St. Louis, MO, USA) at 37°C or at 4°C (negative control) for 2 h. Antigen uptake was determined by flow cytometry from the mean difference in mean fluorescence intensity (ΔMFI), where ΔMFI = MFI (37 °C) – MFI (4°C).

### T Cell Proliferation

Mixed leukocyte reactions (MLR) were carried out to evaluate the relationship between DCs and proliferation of T cells as described (21). DCs were co-cultured with autologous naïve T cells from spleens at a ratio of 1:10 in 96-well plates—all experiments were repeated thrice. All co-cultures in study were performed using PBS, HI and NDV stimulated DCs to further stimulate T cells. After 48 h, T cell proliferation was measured by viability assay using CCK-8 (Dojindo, Kumamoto, Japan) at 37°C for 4 h and stimulation index (SI) was calculated as: SI = (ODsample well-ODblank well)/(ODnegative well-ODblank well).

### Transwell Migration Assay

A transwell system (6.5 mm Transwell^®^ with 8.0 µm Pore Polycarbonate Membrane, Corning) was employed to perform a CCR7-dependent migration assay of infected DCs. DCs were grown in the upper chamber of the Transwell^®^ and the lower chamber was filled with 600 µl complete medium plus CCL19 and CCL21. Migratory cells in the bottom chamber were counted.

### Analysis of Antigen Presentation *In Vivo*

To determine antigen presentation *in vivo*, 6–9-week-old female C57BL/6 mice (6 mice per group) were injected intramuscularly with 100 µl of 10^7.3^ TCID_50_ of NDV. Spleen samples were isolated at 4, 12, 24, 48, and 72 h, and analyzed by flow cytometry.

### Statistical Analysis

All experiments were performed independently at least thrice and results were presented as means ± standard deviation (SD). Student’s t tests were applied to compare the differences between two groups. One-way analysis of variance (ANOVA) was employed to determine significance of differences among multiple groups. Significance levels were defined as *p < 0.05, **p < 0.01, ***p < 0.001, and ****p < 0.0001.

### Ethical Statement

All animal experiments were performed according to the guidelines of the Animal Welfare and Research Ethics Committee of Jilin University (Approval ID: 2016024315-2).

## Results

### Maturation Characteristics of NDV Infected Murine DCs

**Figure 1:**
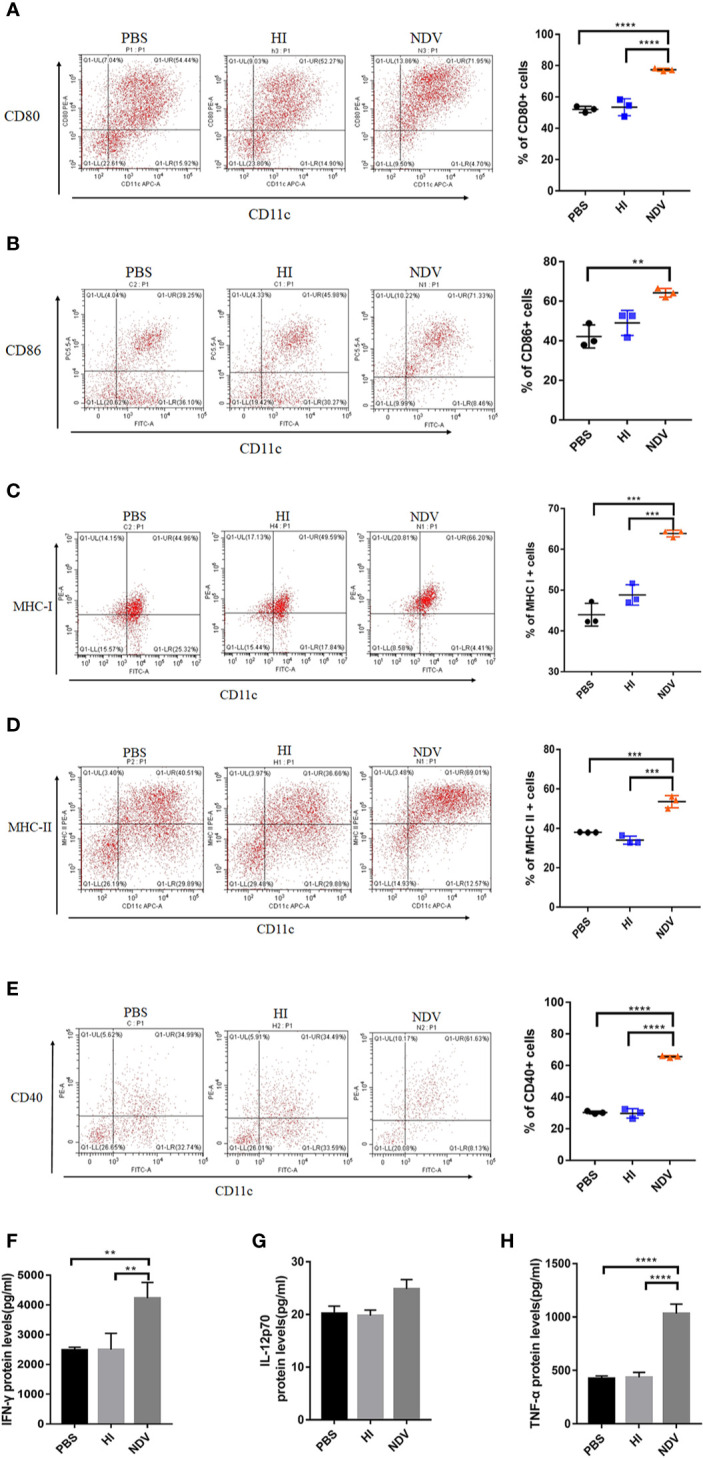
Maturity of DCs mediated by NDV. DCs isolated from murine bone marrow were cultured for 7 days and treated with PBS (mock), HI or NDV at 3 MOI for 48 h respectively. Then flow cytometry was performed to analyze the proportions of CD80 **(A)**, CD86 **(B)**, MHC-I **(C)**, MHC-II **(D),** and CD40 **(E)** expressing DCs. Supernatants of treated DCs were harvested to measure secretion of IFN-γ **(F)**. IL-12p70 **(G)** TNF-α **(H)**. All experiments were performed independently at least thrice and results were presented as means ± standard deviation. Significance levels were defined as **p < 0.01, ***p < 0.001 and ****p < 0.0001.

Since DCs are essential to the initiation of adaptive immunity, the effect of NDV on DCs was evaluated to investigate potential immunomodulatory activity of NDV. imDCs derived from murine BMDCs cultured for 7 days were identified by microscopy and flow cytometry using CD11c-APC or FITC antibodies. The results indicated that the cells were approximately 75% CD11c positive (data not shown) and were morphologically altered (**Figure S1A**)—these were used in subsequent experiments. Compared to PBS or HI treated imDCs, NDV infection significantly enhanced the expression of cell surface molecules including CD40, CD80, CD86, MHC-I, and MHC-II (**Figures 1A–E**). In addition, changes in proinflammatory cytokine secretion (IFN-γ, IL-12p70, TNF-α, IFN-α, and IFN-β) were also demonstrated by ELISA. These cytokines have critical roles in maturation of DCs and differentiation of T cells. The results showed that infection with NDV dramatically upregulated all types of cytokines except IL-12p70 (**Figures 1F–H**, **Figures S1B, C**). To investigate whether NDV could influence the migratory capacity of DC, we assessed CCR5 and CCR7 expressing DCs and exploited the Transwell^®^ system to test migration of DCs *via* the CCR7-CCL19/CCL21 axis. We found that NDV infection elevated expression of CCR5 and CCR7 (**Figure S2A, B**) and infected DCs migrated in a chemokine (CCL19/CCL21) dose-dependent manner (**Figure S2C**), indicating intact migratory capacity.

In summary, upon NDV infection DCs upregulate costimulatory molecule expression, release multiple cytokines and maintain migratory ability, suggesting they are phenotypically mature.

### NDV Suppresses DC-Dependent T Cell Proliferation

**Figure 2:**
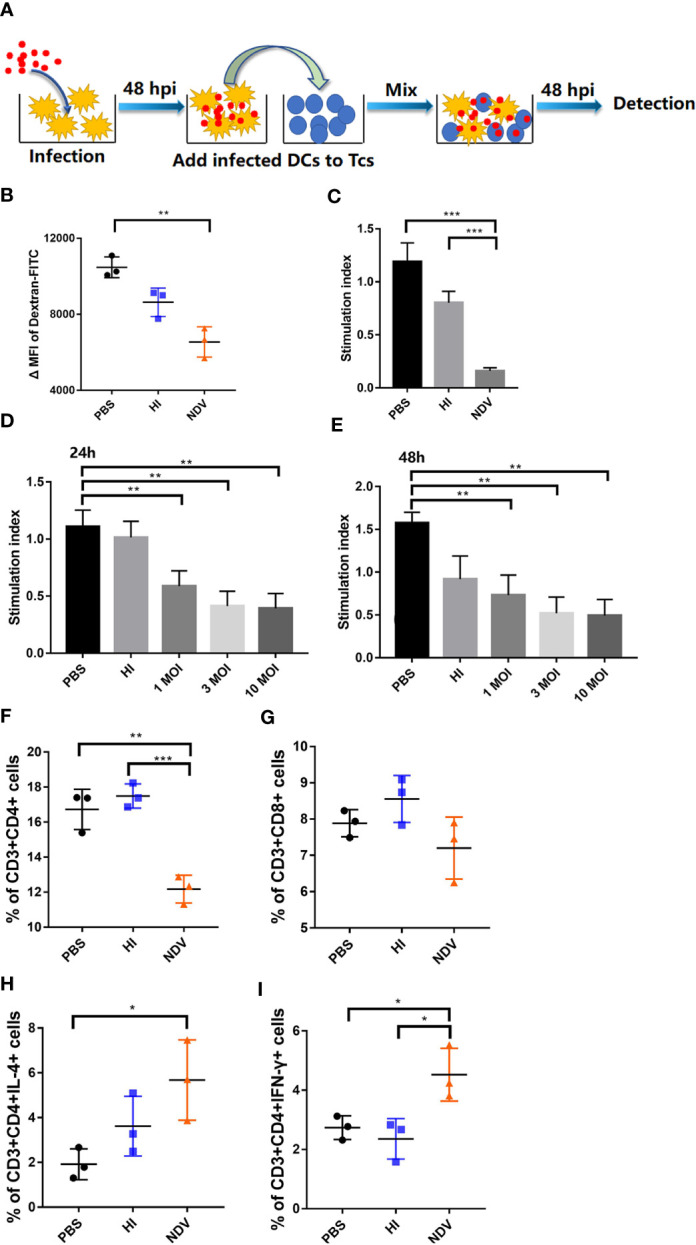
Antigen uptake and presentation of infected DCs. Schematic representation of DC/T-cell co-cultures **(A)**. Treated DCs were incubated with FITC-Dextran at 37°C or 4°C (negative control) respectively and the antigen capture were analyzed by flow cytometry as mean fluorescence intensity value **(B)**. Proliferation of T cells in co-cultures was measured using MLR assay **(C)**. DCs were infected with NDV at 0.1, 1, 10 MOI and co-cultured with T cells. Proliferation of T cells in co-cultures were measured at 24 h **(D)** or 48 h **(E)**. Proportions of CD3^+^CD4^+^ T cells **(F)** and CD3^+^CD8^+^ T cells **(G)** in co-cultures were evaluated by flow cytometry. CD3^+^CD4^+^ T cells secreting IL-4 **(H)** or IFN-γ **(I)** in co-cultures were evaluated by flow cytometry. All experiments were performed independently at least thrice and results were presented as means ± standard deviation. Significance levels were defined as *p < 0.05, **p < 0.01 and ***p < 0.001.

As mature DCs are reported to display reduced antigen uptake capacity and efficient antigen presentation capability (8), FITC-dextran was used in an experiment to assess antigen capture. The ΔMFI value of NDV infected DCs (ΔMFI= 6545) was dramatically lower compared to PBS treated DCs (ΔMFI= 10472), indicating that antigen uptake capability was markedly reduced upon infection (**Figure 2B**). As phenotypic maturation with decreased antigen uptake capacity after NDV infection was confirmed, the question was raised whether infected DCs could effectively activate T cells. DCs were pretreated with PBS, HI or NDV and then collected to further stimulate fresh T cells for 48 h (**Figure 2A**). As shown in **Figure 2C**, NDV treated DCs were weaker in priming T cells than PBS treated DCs. Suppression of T cell proliferation was exacerbated as the viral titer (MOI value) increased (**Figures 2D, E**). We further investigated the DCs’ ability to polarize T cells after infection. In co-cultures, the percentage of CD3^+^CD4^+^ and CD3^+^CD8^+^ T cells in the NDV treated group profoundly decreased after NDV infection (**Figures 2F, G**). However, intracellular IFN-γ and IL-4 in CD4^+^ T cells were elevated—T cells of the CD4^+^ IL-4^+^ (**Figure 2H**) or CD4^+^ IFN-γ^+^ (**Figure 2I**) phenotypes could be activated by NDV treated DCs, especially the CD3^+^CD4^+^IL-4^+^ T cells. Whereas, there were no significant differences in intracellular IFN-γ and IL-4 in CD8+ T cells (**Figures S3A, B**).

Taken together, our data revealed that NDV infection induced a dysfunctional state of DCs, associated with inefficient antigen presentation in DC-T-cell co-cultures.

### NDV Induces Low Levels of Apoptosis in DCs and T Cells

**Figure 3:**
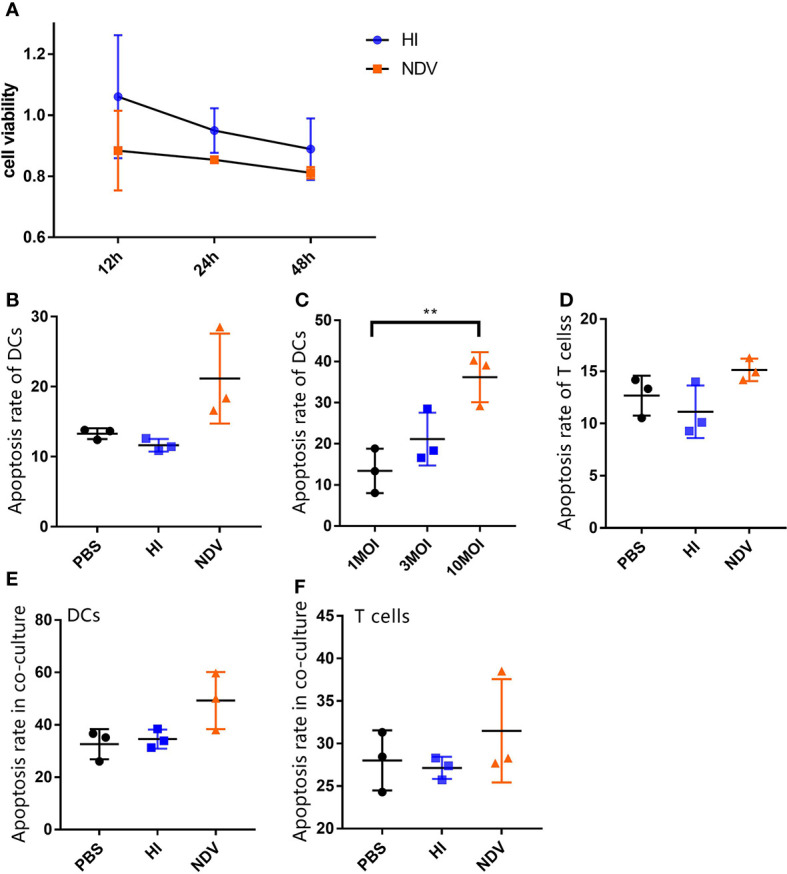
Flow cytometry analysis of apoptosis of DCs and T cells. Cell viability of treated DCs were measured using CCK-8 **(A)**. Treated cells were stained with FITC-annexin V and PI, and then apoptosis rates of infected DCs **(B)** and T cells **(D)** at 3 MOI or indicated MOIs **(C)** were analyzed by flow cytometry. Treated DCs were co-cultured with T cells and then apoptosis rates of DCs **(E)** or T cells **(F)** in co-cultures were measured. All experiments were performed independently at least thrice and results were presented as means ± standard deviation. Significance level was defined as **p < 0.01.

Since we observed a suppressive effect of NDV on DCs with respect to T cell responses, we were interested to determine whether apoptosis was induced directly in T cells or indirectly through modulation of the quality and phenotype of APCs. Upon infection, cell viability decreased by less than 20% compared to PBS at 48 h (**Figure 3A**). Besides this, FITC-annexin V assays revealed that NDV induced only 21% and 15.12% apoptosis in DCs and T cells, respectively, which was slightly higher than the PBS or HI controls (**Figures 3B, D**). Even at 10 MOI, NDV infection-induced apoptosis was no more than 20% higher than in PBS treated DCs (**Figure 3C**). In parallel, we noted that the presence of apoptotic cells within co-cultures was similar to the above results (**Figures 3E, F**). Our observations suggested that the limited apoptosis induced by NDV might not be the critical factor in immunosuppression of T cell proliferation.

### NDV Upregulates Expression of IL-10

**Figure 4:**
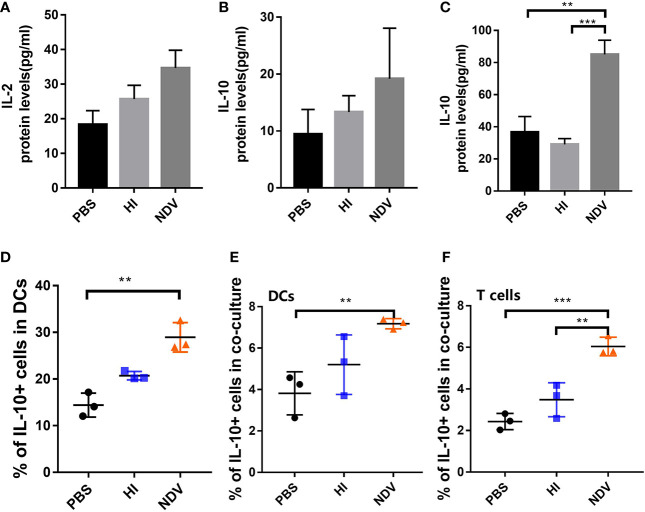
Effect of NDV infection on differentiation of T cells. Supernatants of co-cultures were harvested to measure the secretion of IL-2 **(A)** and IL-10 **(B)** by ELISA. Supernatants of pretreated DCs were collected to measure the secretion of IL-10 **(C)**. Populations of IL-10 expressing DCs were detected using flow cytometry **(D)**. Proportions of IL-10^+^ DCs **(E)** and T cells **(F)** in co-cultures were measured. All experiments were performed independently at least thrice and results were presented as means ± standard deviation. Significance levels were defined as **p < 0.01 and ***p < 0.001.

Differentiation of T cells is influenced by several cytokines. To dissect the polarization of T cells co-cultured with DCs, we measured cytokines including the Th1 proinflammatory cytokine IL-2 and the Th2 immunosuppressive cytokine IL-10, using ELISA. When incubated with NDV, the secretion of IL-2 and IL-10 in co-cultures was higher than with PBS (**Figures 4A, B**). In addition, IL-10 levels in supernatants of NDV-infected DCs were significantly higher than PBS treated DCs at 48 h (**Figure 4C**). In order to evaluate proportions of cells expressing IL-10, DCs and T cells in co-culture were intracellularly stained with IL-10-PE antibody. The results demonstrated that IL-10-expressing cells increased in both NDV-infected DCs and co-cultured cells (**Figures 4D–F**). Generally, IL-10 acts through multiple immunosuppressive mechanisms, mainly affecting the expression of proinflammatory cytokines and chemokines and inhibiting the function of antigen presenting cells (15). Taken together, our results suggested that induction of IL-10 during NDV infection might suppress functional maturation of DCs, resulting in poor T cell priming.

### NDV Facilitates Maturation and Migration of DCs but Inhibits Proliferation of T Cells *In Vivo*

**Figure 5:**
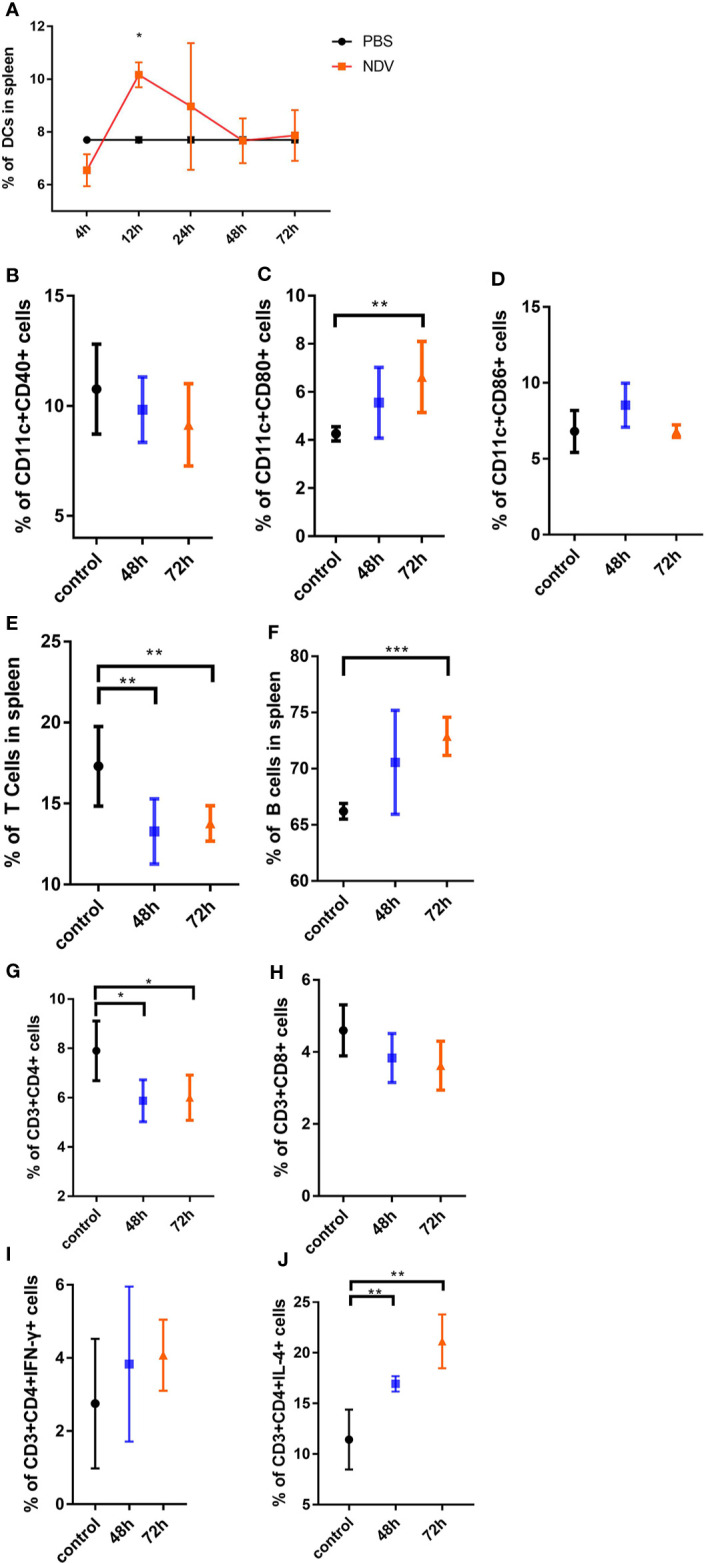
*In vivo* analysis of maturation of DCs and activation of T cells driven by NDV. Mice were inoculated with 100 µl of 10^7.3^ TCID_50_ NDV and the spleen samples were isolated at indicated times. The ratios of DCs from spleens were measured by the specific marker (CD3^-^CD11c^+^ and MHC-II^+^) through flow cytometry analysis **(A)**. The proportions of CD11c^+^CD40^+^ cells **(B)**, CD11c^+^CD80^+^ cells **(C)**, CD11c^+^CD86^+^ cells **(D)** were analyzed by flow cytometry. The portions of CD3^+^ T cells **(E)**, CD19^+^ B cells **(F)**, CD3^+^CD4^+^ cells **(G)**, CD3^+^CD8^+^ cells **(H)**, CD3^+^CD4+ cells secreting IFN-γ **(I),** and CD3^+^CD4^+^ cells secreting IL-4 **(J)** in the spleen were shown. All experiments were performed independently at least thrice and results were presented as means ± standard deviation. Significance levels were defined as *p < 0.05, **p < 0.01 and ***p < 0.001.

Having shown that NDV suppresses functional maturation of DCs and their CD4^+^ T cell priming capacity *in vitro*, we sought to determine the immune stimulatory effect of NDV *in vivo*. C57BL/6 mice were injected with NDV in the thigh muscle of the hind leg, and spleen samples were collected at indicated time points for DC and T cell analysis. We took advantage of CD11c and MHC-II coexpression as a specific marker of DCs to monitor the dynamics of DCs in the spleen. Analysis of splenic populations showed that CD11c^+^ MHC-II^+^ DCs were depleted to a nadir (6.55%) early after infection, increased and reached a peak (10.17%) at 12 h, but stabilized thereafter to 7.7% at 48 h (**Figure 5A**). Additionally, NDV recruited subsets of CD11c^+^ CD86^+^ and CD11c^+^ CD80^+^ DCs into the spleen (**Figures 5C, D**). However, CD11c+ CD40+ DCs were decreased (**Figure 5B**). Consistent with the *in vitro* data, CD3^+^ CD4^+^, and CD3^+^ CD8^+^ T cell populations decreased when NDV was injected (**Figures 5G, H**) and the proportions of CD3^+^ CD4^+^ IFN-γ^+^ and CD3^+^ CD4^+^ IL-4^+^ T cells increased (**Figures 5I, J**), especially the latter which were significantly elevated compared to PBS (*P*< 0.001). Compared to this marked diminution of T cells (**Figure 5E**), the proportion of B cells (CD19^+^) in spleens rose strikingly (**Figure 5F**), indicating a predominant activation of Th2 cells and B cells upon NDV infection *in vivo*. Taken together, the *in vivo* data reveal that antigen presentation of NDV antigens occurs mainly from 4 h to 72 h and the response to NDV results in B cell biased proliferation and predominant Th2 differentiation.

## Discussion

DCs are professional antigen presenting cells which are of vital importance in concatenating innate and adaptive immune responses. Functional maturation of DCs is characterized by decreased antigen capture and efficient antigen presentation (22). Previous studies in the context of NDV have shown that although the expression of surface markers and cytokines is efficiently enhanced (3, 4), DCs may remain non-functional as a consequence of apoptosis (5). In this study, *in vitro* results indicated that infective NDV acted as a potent inducer of DC maturation, accompanied by increased expression of MHC-I, MHC-II, CD40, CD80, and CD86 on the cell surface (**Figures 1A–E**), as well as the production of pro-inflammatory cytokines (**Figures 1F–H**). Additionally, infected DCs displayed decreased antigen uptake (**Figure 2B**) and unchanged migratory capacity (**Figure S2C**) providing sufficient evidence for phenotypic maturation. However, despite this maturity, DCs were not capable of stimulating T cells (**Figures 2C–E**). When co-cultured with pretreated DCs, T cell populations exhibited decreased numbers of CD3^+^CD4^+^ and CD3^+^CD8^+^ cells with a predominant activation of Th2 cells. These data are consistent with the work of Sousa revealing expression of costimulatory molecules on DCs may not correlate with functional maturation (22). Generally, the ability of DCs to present viral antigens to T cells is critical in generating an effective adaptive immune response. Therefore, attenuation of DC antigen presentation might be a strategy exploited by NDV to compromise the adaptive immune response of the host in order to prolong viral persistence and hence increase the chances of horizontal transmission. DCs appeared to mature phenotypically in response to NDV infection - nevertheless, the ability of infected cells to active naïve T cells was compromised.

To suppress DC/T cell crosstalk during infection, viruses exert pleiotropic suppressive effects towards diverse cells involved in innate and adaptive immune responses. Increased apoptosis of both DCs and T cells influences their functionality, leading to decreased stimulatory capability of virus-infected DCs (8). For example, MV infection results in upregulation of Fas and TRAIL-mediated apoptosis in DC/T-cell co-cultures, which contributes to T cell lysis (23, 24). Surprisingly therefore, the proportion of apoptotic infected cells we observed seemed low, even with a high viral input (10 MOI) or long duration of infection (**Figures 3B–F**), which might not be sufficient to constrain NDV-specific T cell responses. Grosjean et al. argued that the 25% apoptotic cells they observed in co-cultures could not be responsible for the extent of immunosuppression (25). In contrast to our results, high levels of apoptosis in DCs in the context of infection with the Herts/33 strain of NDV has been reported (5). However, Herts/33 is a velogenic strain associated with higher pathogenicity than the LaSota lentogenic strain.

An IL-10-dominated immunosuppressive environment would negatively affect the size and quality of the adaptive immune response. MCMV can induce IL-10 to dampen innate immune responses and attenuate DC function, eventually resulting in impaired CD4^+^ T cell priming (16). Viruses associated with chronic infection often exploit the IL-10 pathway to modulate host antiviral immunity to viral replication. In our study, infected DCs and co-cultures appeared to express higher concentrations of IL-10 than PBS-treated DCs (**Figures 4B–F**), which might shape the potency of DCs, subsequently affecting T cells priming. Moreover, IL-10-expressing Th2 CD4^+^ cells suppress the ability of DCs to prime Th1-polarized CD4^+^ T cells (26).

Maturation shifts the function of DCs from a primary role of patrolling the periphery to migration towards secondary lymphoid organs and processing and presentation of antigens *via* MHC to T cells (20). To assess antigen presentation of NDV by DCs *in vivo*, we performed analysis and surveillance of activation of DCs and T cells in the spleen. The observation that merits attention is that DCs migrated to the infection site early upon injection (4 h), then decreased in the subsequent 24 h to 72 h, which might be attributed to priming of B cells (**Figures 5A, E, F**). Therefore, antigen presentation of NDV *in vivo* mainly occurred from 4 h to 72 h. The type of Th cell differentiation is typically determined by strength of stimulation. Specifically, weak stimulation drives uncommitted cells, moderate stimulation leads to Th2 differentiation, while strong stimulation induces Th1 differentiation (27). We also assessed whether intramuscular injection of NDV could lead to T cell suppression. As expected, the results showed decreased rather than increased proportions of virus-specific CD3^+^CD4^+^ and CD3^+^CD8^+^ T cells *in vivo* (**Figures 5G, H**). Conversely, compared to the restrained T cell populations, proportions of B cells in spleen were markedly augmented after infection with NDV. Moreover, in the presence of NDV, T cells secreted more IFN-γ and IL-4 (especially IL-4), which is involved in promoting the proliferation of Th2 cells (**Figure 5J**). Increased Th2 cells inhibit Th1 cell proliferation and are crucial for the priming of B cells (28).

In conclusion, we have investigated the effect of NDV on DCs during antigen presentation, and found it has a suppressive impact on priming of DC-mediated T cell responses. Infection of DCs was associated with enhanced expression of surface markers and proinflammatory cytokines, while inhibiting proliferation of T cells through IL-10. Furthermore, antigen presentation by DCs during the early phase of infection *in vivo* depleted populations of T cells and promoted Th2 immune responses, consequently leading to B cell proliferation. Our data illustrate that NDV has devised a strategy to dampen the proliferation of T cells *via* IL-10-associated Th2 immune responses, mediated through DCs. This represents a novel pathway used by NDV to suppress adaptive immunity to its own advantage.

## Data Availability Statement

The raw data supporting the conclusions of this article will be made available by the authors, without undue reservation.

## Ethics Statement

The animal study was reviewed and approved by Animal Welfare and Research Ethics Committee of Jilin University (Approval ID: 2016024315-2).

## Author Contributions

NJ, HL, YZ, FN, and WZ were responsible for experiment design and drafting the manuscript. FN, WN, TY, CX, HZ, XX, CL, YZ, ZH, JZ, XZ, JH, WW, JQ, GZ, ZL JG, and ZB performed experiments and analyzed data. All authors contributed to the article and approved the submitted version.

## Funding

This work was supported by grants from National Natural Science Foundation of China (No. 31272573).

## Conflict of Interest

The authors declare that the research was conducted in the absence of any commercial or financial relationships that could be construed as a potential conflict of interest.
