# Supplementary material for: Newcastle Disease Virus Inhibits the Proliferation of T Cells Induced by Dendritic Cells In Vitro and In Vivo
Source: Front Immunol. 2021 Feb 23;11:619829. doi: 10.3389/fimmu.2020.619829 (PMC7942023; doi:10.3389/fimmu.2020.619829)
Supplement: Supplementary file 1 [file DataSheet_1.docx]

***Supplementary Material***

## Supplementary Figures


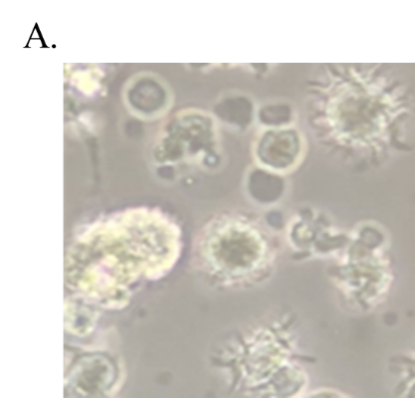

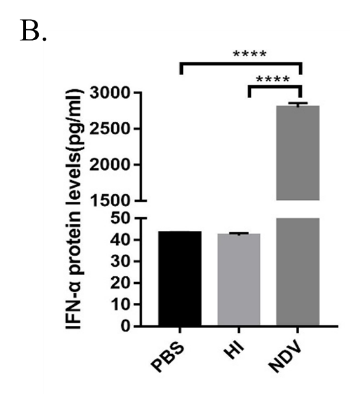

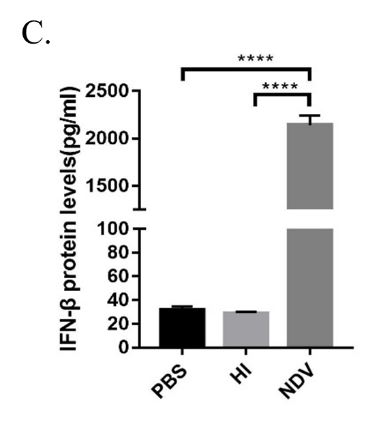


**Supplementary Figure 1 |** DCs phenotypically matured in the context of NDV. Leading uropod on surface of imDCs were observed at day 7 **(A)**. Supernatants of treated DCs were harvested to measure the secretion of IFN-α **(B)** and IFN-β **(C)** by ELISA. All experiments were performed independently at least thrice and results were presented as means ± standard deviation. Significance level was defined as ****p < 0.0001.


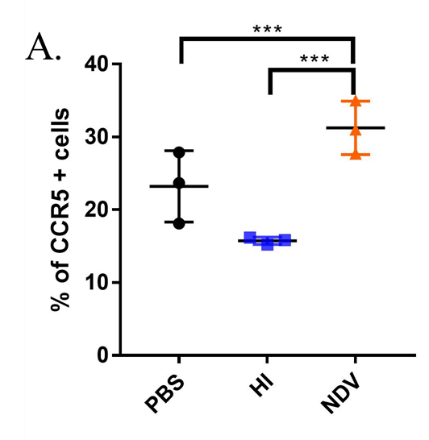

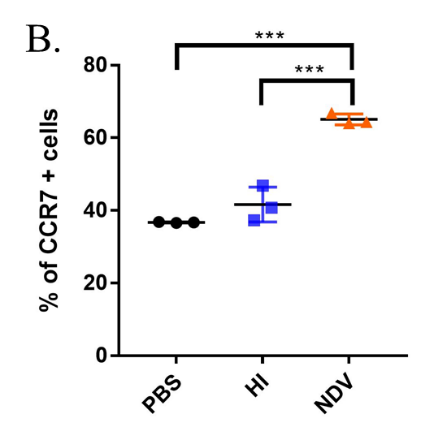


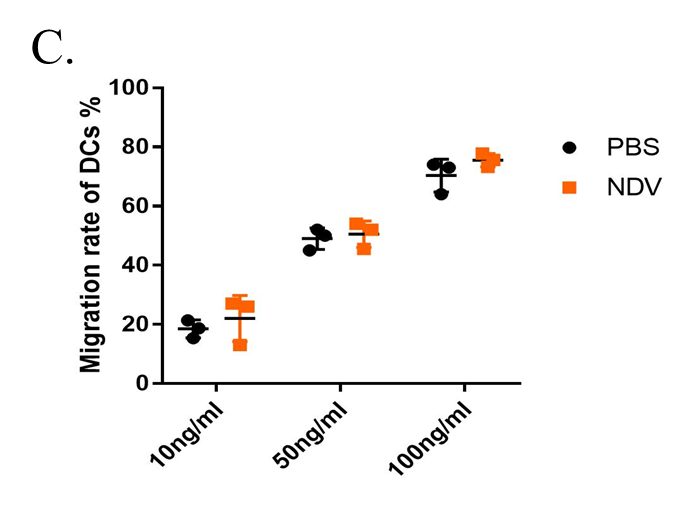


**Supplementary Figure 2 |** Migration of infected DCs through CCR7-CCL19/CCL21 axis. Flow cytometric analysis of CCR5 **(A)** and CCR7 **(B)** expressing DCs. Transwell® system was exploited to detect the portion of migratory DCs. DCs placed in the upper chamber were migrated through an increasing dose of CCL19 and CCL21 in the lower chamber **(C)**. All experiments were performed independently at least thrice and results were presented as means ± standard deviation. Significance level was defined as ***p < 0.001.


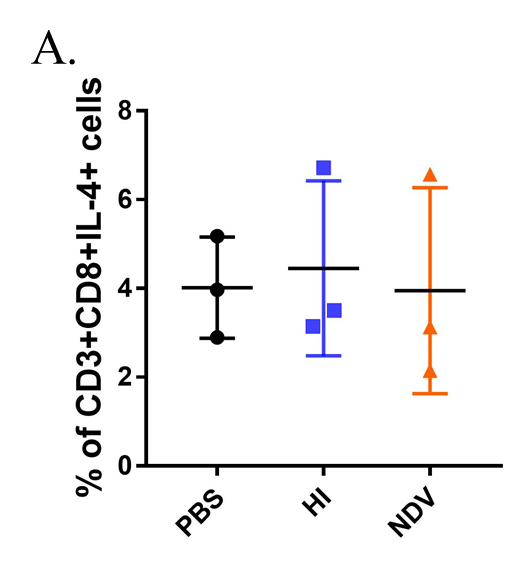

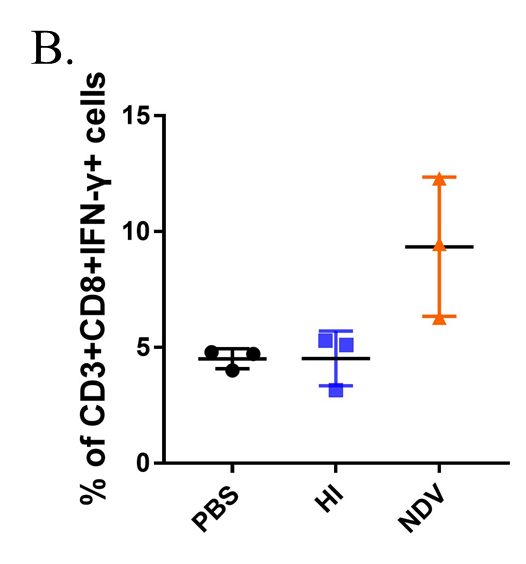


**Supplementary Figure 3 |** Activation of CD3^+^CD8^+^ T cells in co-cultures. DCs were infected with NDV at 3 MOI and then cocultured with T cells. CD3^+^CD8^+^T cells secreting IL-4 **(A)** and IFN-γ **(B)** in cocultures were evaluated by flow cytometry. All experiments were performed independently at least thrice and results were presented as means ± standard deviation.
